# Evaluation of image-guided and surface-guided radiotherapy for breast cancer patients treated in deep inspiration breath-hold: A single institution experience

**DOI:** 10.1016/j.tipsro.2022.02.001

**Published:** 2022-02-17

**Authors:** Joan Penninkhof, Kimm Fremeijer, Kirsten Offereins-van Harten, Cynthia van Wanrooij, Sandra Quint, Britt Kunnen, Nienke Hoffmans-Holtzer, Annemarie Swaak, Margreet Baaijens, Maarten Dirkx

**Affiliations:** Department of Radiation Oncology, Erasmus MC Cancer Institute, Rotterdam, The Netherlands

**Keywords:** Breath-hold, Surface-guided radiotherapy, DIBH, Breast, DIBH, Deep inspiratory breath-hold, SGRT, surface-guided radiotherapy, DRRs, digitally reconstructed radiographs, CT, computer tomography, CBCT, cone-beam CT, OTM, online treatment monitor, (U, V), ventral-dorsal and cranial-caudal direction in the tangential beam, respectively, NAL, no-action-level setup correction protocol, eNAL, extended NAL setup correction protocol, VRT, anterior-posterior direction, LNG, cranial-caudal direction, LAT, medio-lateral direction

## Abstract

**Introduction:**

Nowadays, deep inspiratory breath-hold is a common technique to reduce heart dose in left-sided breast radiotherapy. This study evaluates the evolution of the breath-hold technique in our institute, from portal imaging during dose delivery to continuous monitoring with surface-guided radiotherapy (SGRT).

**Materials and methods:**

Setup data and portal imaging results were analyzed for 98 patients treated before 2014, and SGRT data for 228 patients treated between 2018 and 2020. For the pre-SGRT group, systematic and random setup errors were calculated for different correction protocols. Residual errors and reproducibility of breath-holds were evaluated for both groups. The benefit of using SGRT for initial positioning was evaluated for another cohort of 47 patients.

**Results:**

Online correction reduced the population mean error from 3.9 mm (no corrections) to 1.4 mm. Despite online setup correction, deviations greater than 3 mm were observed in about 10% and 20% of the treatment beams in ventral-dorsal and cranial-caudal directions, respectively. However, these percentages were much smaller than with offline protocols or no corrections. Mean absolute differences between breath-holds within a fraction were smaller in the SGRT-group (1.69 mm) than in the pre-SGRT-group (2.10 mm), and further improved with addition of visual feedback (1.30 mm). SGRT for positioning did not improve setup accuracy, but slightly reduced the time for imaging and setup correction, allowing completion within 3.5 min for 95% of fractions.

**Conclusion:**

For accurate radiotherapy breast treatments using deep inspiration breath-hold, daily imaging and correction is required. SGRT provides accurate information on patient positioning during treatment and improves patient compliance with visual feedback.

## Highlights

• SGRT provides continuous non-invasive monitoring of patient’s positioning and DIBH.
• Variation in positioning and DIBH requires daily online setup corrections.
• Setup using SGRT is slightly faster than laser-based setup, with similar accuracy.
• Visual feedback to the patient enhances DIBH compliance and reproducibility.

## Introduction

Radiation therapy after lumpectomy has become the standard treatment for breast cancer patients. It reduces the risk of local recurrence with a factor of 3–4 compared to no radiotherapy [1]. Improvements in screening, local and systemic treatments have led to improved survival in all breast cancer patients, especially in low-risk groups. Quality of life and long-term radiation-related toxicity, such as cardiotoxicity and breast fibrosis, are therefore of major concern. Modern radiotherapy techniques enable dose reduction to the heart without compromising the dose coverage to the target. Nowadays, the most commonly applied technique for cardiac sparing is deep inspiration breath-hold (DIBH) [2]. When a patient inhales deeply, the volume of the lungs increases, and the heart is pushed down and away from the chest wall and breast volume to be treated. This allows for a reduction of dose to the heart and its substructures compared to treatment in free breathing [3], [4].

To ensure correct dose delivery at the treatment unit, several systems have been developed and commercialized in the past decades to support and monitor the depth and reproducibility of successive breath-holds during dose delivery [5], [6], [7]. These systems are either invasive (e.g., spirometer-based) or non-invasive (e.g., optical surface-guidance systems, pressure belts or external markers). Portal imaging can be used to evaluate the dose delivery during DIBH as well e.g., [8], [9], [10], [11], [12]. Even with low-cost visual monitoring of the light field projection of the treatment field or positioning lasers against reference skin marks [13], DIBH reproducibility was shown to be comparable to that with a spirometry-based device, and superior in terms of patient acceptability and ease of implementation [14]. As all techniques reduce dose exposure to the heart, the choice of DIBH method is largely up to the institution and will most likely be based on existing equipment and experience [7]. External monitoring devices may be combined with gating techniques; the gating software will automatically detect when to deliver radiation (i.e., in case the patient's breath-hold is in tolerance), or when the beam must be held (e.g., disruptions in breath-hold, coughing or sneezing).

For breast radiotherapy, surface-guided radiation therapy (SGRT) has shown to be beneficial for patient positioning, patient monitoring throughout the treatment fractions, gating in free breathing or DIBH, and detecting anatomical variations throughout the treatment course [15], [16], [17], [18], [19], [20], [21], [22]. The optical surface scanners provide imaging of the patient’s skin position with high spatial and temporal resolution. Surface-guided correction of the patient posture, arm and chin position also improves the position of the breast in general. An SGRT workflow may therefore reduce time required for image registration, and has the potential to reduce the imaging frequency and additional imaging dose [16]. A tattoo-free radiotherapy workflow for accelerated partial breast treatments has recently been reported [23], which will improve patient quality‐of‐life without sacrificing treatment accuracy.

The radiotherapy department of the Erasmus MC Cancer Institute treats more than 5500 patients on a yearly basis; about 20% of the patients are treated for breast cancer. Since 2012, DIBH treatment has been offered to left-sided breast cancer patients. Initially, the depth of breath-hold was monitored by real-time portal imaging of the treatment beams, overlaid with reference structures from the digitally reconstructed radiographs (DRRs). In 2018, this system was replaced by a surface guidance system, enabling monitoring during patient positioning and treatment dose delivery. In this study, we summarize the clinical experience with DIBH in our institute, and describe the evolution of our DIBH technique since early 2012. Data of two large cohorts, representing the portal imaging monitored and the SGRT-monitored DIBH treatments, were analyzed. For the early DIBH cohort (pre-SGRT), the impact of image-guided position correction protocols on the residual errors was evaluated. The initial set-up with SGRT and the use of a visual feedback system was evaluated in subgroups of the SGRT patient cohort.

## Materials and methods

### Patient positioning and general workflow

Patients under 70 years with left-sided breast cancer were eligible for DIBH, both after lumpectomy and mastectomy. Although patient monitoring evolved over the years, patient positioning and workflow remained almost the same. About 3–5 days prior to planning computer tomography (CT) acquisition, all patients received an individual training and were coached to hold a stable DIBH for 35–40 s. CT-scans in DIBH and free breathing were acquired in supine position on a breast board at an angle of 10 degree with both arms raised over the head and positioned on an arm-support device. At the treatment unit (Elekta AB, Stockholm, Sweden), the patient was positioned by aligning the tattoos with the laser-system in free breathing, after which a relative couch shift was applied to obtain the treatment isocenter in DIBH. Patient positioning was daily verified and corrected before delivery of the actual treatment beams by using a 180 degree cone-beam CT acquisition in DIBH (CBCT, XVI, Elekta AB, Stockholm, Sweden) and registration on the thoracic wall and breast contour. The total time per treatment fraction was 12–16 min. About 10–15% of the patients were not treated in DIBH, because they could not hold a stable DIBH, or were being treated with arc therapy or protons.

### Treatment planning

Over the years, different treatment planning techniques were applied according to protocol in either the XiO or Monaco treatment planning system (Elekta AB, Stockholm, Sweden). All techniques were based on tangential opposing fields and planned on the DIBH planning CT: (1) a conformal planning technique, (2) a forward-planned field-in-field technique, or (3) a hybrid technique in which open beams deliver 70–80% of the dose and segments derived with inverse optimization deliver the remainder [24]. For all techniques, treatment plans generally consisted of four beams, with a maximum of eight in case of axilla involvement [11]. The number of monitor units per beam was restricted, so that the beam could be delivered within 25 s. In general, the delivery time per beam was between 12 and 18 s. Beams with energies of either 6 or 10 MV were used, or in combination, depending on the patient’s anatomy. Patients were treated either with a conventional fractionated (2 Gy in 25 fractions) or a hypofractionated schedule (2.66 Gy in 16 fractions, or 2.67 Gy in 15 fractions). When indicated, a sequential boost to the tumor bed was applied in 5 – 8 fractions.

### Pre-SGRT: Monitoring with portal imaging only

Until 2018, breath-hold stability and intra-fraction setup between successive breath-holds was verified by continuous imaging during delivery of the radiation beams using an electronic portal imaging device. In the Theraview-NT software (Cablon Medical, Leusden, the Netherlands), we used the online treatment monitor (OTM), a dedicated breath-hold module in which the breast and lung contours from the DRRs were projected real-time on top of the individual portal imaging frames (what you see is what you treat). Dose delivery was manually interrupted for chest wall setup deviations larger than 4 mm. The stability of the position of the thoracic wall within each DIBH could be evaluated in the movie frames [9]. For each treatment field, the average of all frames in a movie was calculated and stored as a setup image. All setup images for tangential beams were registered off-line to the reference DRRs, by matching on the caudal part of the breast (cranial-caudal direction, V) and the lung/thoracic wall contour (ventral-dorsal direction, U).

### Transition from OTM to SGRT

**Fig. 1:**
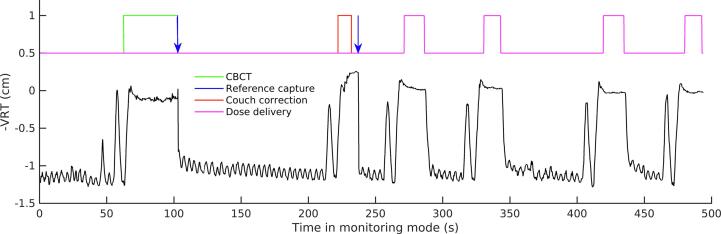
Surface guided radiotherapy (SGRT) workflow for deep-inspiration breath-hold since May 2018. SGRT signal for the movement in ventral-dorsal direction is shown in time for one fraction. During the imaging procedure (t = 50–235 sec), reference surfaces are captured (blue arrow) at the end of CBCT acquisition (indicated in green) to link the external surface to the internal anatomy on the CBCT, and after couch movement for setup correction (red). Afterwards, dose was delivered with four treatment beams (magenta). Prior to each DIBH, the patient was asked to deeply breathe in and out once. (For interpretation of the references to colour in this figure legend, the reader is referred to the web version of this article.)

In 2018, the real-time OTM-based workflow was replaced by a surface guided radiotherapy (SGRT) workflow using the optical surface monitoring system AlignRT (VisionRT Ltd. London, UK). This system consists of three ceiling-mounted cameras, and allows for continuous monitoring of the patient’s skin surface during the full time-span of treatment. In addition, a visual feedback system (real time coach, clinically introduced in 2020) can be offered to the patient, displaying the breathing motion in ventral-dorsal direction. All patients were treated with a combination of CBCT and SGRT. The clinical workflow (Fig. 1) includes a capture of a reference surface at the end of the CBCT acquisition (when none of the cameras are blocked) to link the internal anatomy on CBCT to the actual surface position. After registration of the CBCT, the patient is asked to breathe in to the reference position to apply the on-line setup correction and capture a new reference surface-of-the-day for treatment. During treatment, the applied SGRT tolerances are 3 mm for translations in the orthogonal directions, 3 degrees for rotations and 5 mm for the vector. The region of interest for surface tracking includes the left breast only.

### SGRT-based patient setup

Since early 2021, the SGRT system is also used for initial positioning, both in free breathing (at mid-patient reference point) and in DIBH (at treatment isocenter). The region-of-interest selected during positioning includes both breasts, while monitoring during DIBH treatment is done on the left breast only.

### Patient cohorts and analyses

In the pre-SGRT patient cohort, online portal imaging was used to monitor DIBH. Patients in this cohort were treated between January 2012 and July 2014. The residual error in the 2D plane of the medio-lateral beam was determined from registration of the setup images of the tangential beams (98 patients, 8050 beams, 2450 fractions). To evaluate the impact of correction protocols on the setup errors, the clinically applied online corrections were made undone, and population systematic and random setup errors were simulated for the situation that no setup correction protocol, an offline no-action-level (NAL N = 3) or an offline extended NAL (eNAL) protocol [25] would have been applied.

For the SGRT-monitored cohort, DIBH patients treated between May 2018 and October 2020 were retrospectively selected (SGRT cohort). The log files from the SGRT database include the deviation from the reference surface for each frame in time, for three orthogonal translations and rotations (i.e., anterior-posterior (VRT), cranial-caudal (LNG) and medio-lateral (LAT) directions). By combining this information with the treatment logs from the record-and-verify system, the average setup error and stability of each DIBH was derived. For this cohort, 14,252 beams were analyzed (228 patients).

Prior to the implementation of SGRT for monitoring DIBH, from January until May 2018, a small prospective study with 19 patients (not included in any other cohort) was conducted for cross-validation of the SGRT workflow with the clinically used pre-SGRT workflow (Transition-cohort). As the OTM was clinically used, the level of DIBH was not explicitly monitored using SGRT. For this cohort, both residual setup errors from the portal setup images and the SGRT system logs were analyzed (1080 beams).

Clinical evaluation of the benefit of the visual feedback system was performed in the first months of 2020 for 10 of the 228 patients included in the SGRT cohort. As a measure for DIBH reproducibility, the difference in residual error in ventral-dorsal direction was used.

The benefit of SGRT for positioning was quantified using the clinically used CBCT registrations (rotations and translations) and the time needed to complete the imaging procedure (including couch movement) for a cohort treated in 2021. In this evaluation, 47 DIBH patients (with SGRT-setup) and 25 DIBH patients (setup based on tattoos only) were included. Using the Shapiro–Wilk test, we confirmed that the observed rotations and translations were normally distributed. The *t*-test was used to evaluate statistical significance with a significance level of 0.05. For differences in imaging time, a non-parametric Wilcoxon rank sum test was used (p = 0.05).

## Results

### Monitoring with portal imaging (pre-SGRT)

**Fig. 2:**
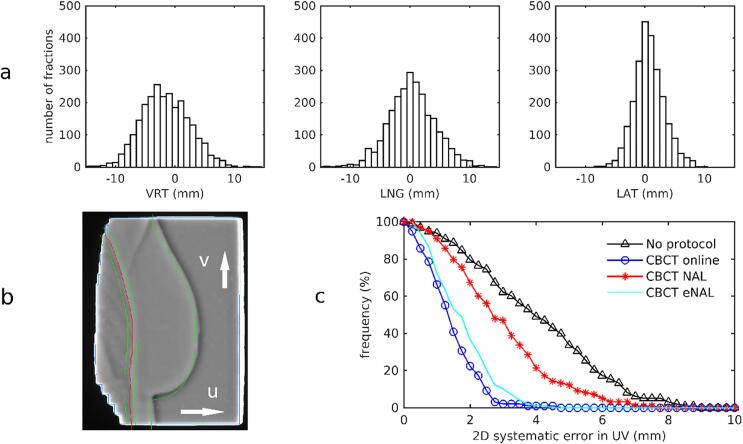
A. Histograms of the applied online cone-beam CT corrections in the pre-SGRT group, in anterior-posterior (left), cranial-caudal (middle) and medio-lateral (right) directions. B. Online treatment monitor, showing the projection of the breast contour (green) and the lung contour ± 4 mm margin (red and green) from the DRRs on top of individual, real-time acquired portal imaging frames. In this case, image registration revealed a residual offset after online CBCT correction of −4 mm in cranial-caudal direction (V). c. Cumulative histogram of the residual 2D systematic setup error projected on the UV-plane of the medio-lateral beam: without correction protocol (black triangles), with online corrections (blue circles), with a no-action-level protocol (NAL N = 3, red stars), and with the extended NAL protocol (cyan line). (For interpretation of the references to colour in this figure legend, the reader is referred to the web version of this article.)

The top panel in Fig. 2 shows the distribution of the clinically applied online CBCT corrections in VRT, LNG and LAT directions for the pre-SGRT cohort. Mean ± 1 SD values over all fractions and patients were −1.54 ± 4.01 mm in VRT, 0.36 ± 3.90 mm in LNG, and 0.75 ± 2.51 mm in LAT, respectively. The residual errors, determined from registration of the setup images of the tangential beams, were used to calculate the 2D systematic error in the plane of the medio-lateral beam. The cumulative distribution is shown in Fig. 2. In this figure, the simulated distributions for no correction and the offline NAL and eNAL protocols are shown as well. Without setup correction, *systematic* 2D errors of 5 mm or more would have occurred in 33% of the patients, while with an online protocol 98% of the *systematic* errors were smaller than 3 mm. By using online CBCT corrections, the population mean error reduced from 3.9 mm (no corrections) to 1.4 mm. Without correction, the *random* error without corrections was 2.3 mm in ventral-dorsal direction (U) and 2.7 mm in cranial-caudal direction (V); this was reduced to 1.3 mm in U and 1.6 mm in V after online CBCT correction.

When evaluating simulated displacements for the individual beams, protocols showed differences in U larger than 5 mm for 23% (no protocol), 14% (NAL), 11% (eNAL) of the beams compared to 1.5% in case of online corrections. In V-direction, these numbers were 22% (no protocol), 21% (NAL), 15% (eNAL), compared to 5.1% (online). This clearly shows that, in spite of the considerable reduction in systematic error with eNAL compared to NAL, both offline protocols are not capable to reduce the variability in DIBH and positioning for this patient group.

### Transition from OTM to SGRT

**Fig. 3:**
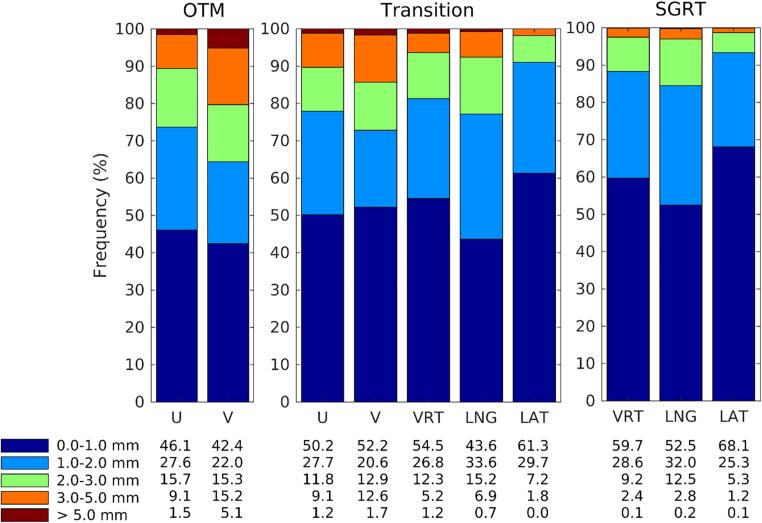
Frequency (%) of detected deviations larger than 1, 2, 3, and 5 mm in ventral-dorsal (U) and cranial-caudal direction (V) in 98 OTM-monitored patients, and in anterior-posterior (VRT), cranial-caudal (LNG) and medio-lateral (LAT) directions in 228 SGRT-monitored patients. For a study-group of 19 patients during the transition period, both data is shown.

Fig. 3 shows the residual errors per treatment beam, classified in 1-mm-bins. The left panel of Fig. 3 depicts results in U and V direction of the portal imaging in the pre-SGRT group. 42–46% of the DIBHs is within 1 mm of the reference in U or V direction. Despite online setup correction, about 10% and 20% of the beams still deviate more than 3 mm for the repeated DIBH in U and V, respectively. For the 19 patients in the transition period (middle panel), the OTM was leading, but SGRT-data was collected as well. Similar distributions in (U, V)-directions are observed as in the OTM-group, but SGRT deviations appeared to be smaller than the observed deviations from portal imaging. The average differences between portal imaging and SGRT for the 493 matching beams were −0.38 ± 2.6 mm (1 SD) in U and −0.71 ± 2.0 mm in V, but differences of more than 4 mm were observed as well (data not shown). For the SGRT group (right panel in Fig. 3), more than 85% of beams were delivered with a residual error smaller than 2 mm, and only 3% of the beams deviated more than 3 mm.

**Fig. 4:**
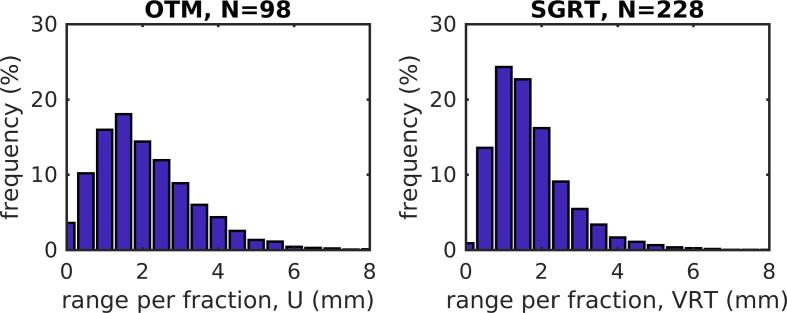
Deep inspiration breath-hold reproducibility in ventral direction improves with surface guidance. Maximum variation in deep inspiration breath-hold over all beams per fraction in ventral direction from portal imaging (OTM cohort, left) and surface guidance (SGRT cohort, right).

As a measure for DIBH reproducibility, differences in ventral direction between beams (U in portal imaging, VRT in SGRT data) for each fraction are depicted in Fig. 4. Mean absolute differences were smaller in the SGRT-group (1.69 mm) than in the OTM-group (2.10 mm). The reproducibility of the DIBH further improved with the addition of the visual feedback system (1.30 mm).

### SGRT-based patient setup

**Fig. 5:**
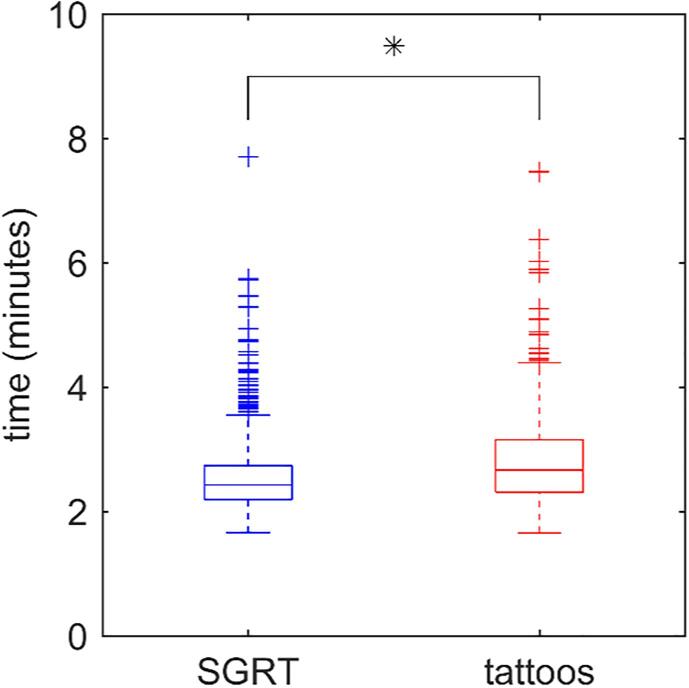
Time needed to complete the imaging procedure (CBCT acquisition, matching and on-line setup correction) for patients positioned on tattoos only (red, 25 patients) or using SGRT (blue, 47 patients). All patients were treated for left-sided breast cancer in deep inspiration breath-hold. * denotes a significant difference based on a Wilcoxon rank sum test (p-value < 0.01). (For interpretation of the references to colour in this figure legend, the reader is referred to the web version of this article.)

Fig. 5 shows the time to complete the imaging procedure, including couch movement, for patients’ setup based on tattoos and setup using the SGRT system. The total imaging procedure lasted 168.7 ± 44.0 s without SGRT-setup, and 152.8 ± 33.2 s with SGRT-setup. Although the reduction is 16 s only, the SGRT-setup could be completed within 3.5 min for 95% of the fractions (770/811). Without SGRT-setup, this was only the case for 85% of the fractions (445/524). Comparison of the online corrections (rotations and translations) between treatments with and without SGRT-setup revealed small differences, not considered *clinically* significant (see supplementary data).

## Discussion

In this manuscript, two workflows to monitor the level of DIBH during breast cancer treatments were evaluated: a workflow using portal imaging and OTM, and a workflow based on surface monitoring. In the latter, the patient’s surface is used as a surrogate for the internal anatomy. Whereas the OTM-based procedure only provides information during dose delivery (see what you treat), with SGRT the patient positioning is monitored during the full treatment fraction. In our study, deviations from reference position per beam were smaller with SGRT than with OTM (Fig. 3), and resulted in a more reproducible DIBH during each fraction (Fig. 4).

Hamming et al [26] showed that differences in setup errors between SGRT and CBCT (registered on skin surface) were within 4.7 mm for 95% of the measurements. Lower correlation was found in longitudinal direction, which was attributed to differences in reference structure: SGRT is mainly probing the ventral side of the breast whereas CBCT registration merely focuses on the caudal side of the breast. In our SGRT workflow, the internal anatomy (as visualized on CBCT) is linked to the external surface by taking a reference SGRT capture at the end of CBCT acquisition. The actual surface reference for dose delivery, however, is taken after applying the online setup correction determined by registration of the CBCT to the planning CT. This may limit the accuracy of the treatment, as the patient has to repeat the DIBH to the same level as during CBCT before the couch is moved to the new position and the reference-of-the-day is captured.

During the transition period from OTM to SGRT, cross-validation of the surface monitoring system with portal imaging was done. The different use of references between the OTM and SGRT workflows may have contributed to the larger residual setup errors in the setup images compared to the SGRT results (Fig. 3). As, in the latter, the patient surface after applying the CBCT acquisition and setup correction is used as the reference, a near perfect registration to the internal anatomy (based on CBCT) and on the patient surface (based on SGRT) is obtained. Additionally, inherent difficulties encountered in planar portal image registration (most pronounced in the V-direction) contribute to the observed differences. In the clinical workflow, a single person performed the image registration. Repeating the registration at a different time or by a different observer might have resulted in slightly different results, as intra- (and inter-)observer variability is 1–2 mm [27].

Our study shows that, due to the relatively large inter-fraction variability for DIBH, online setup correction is required. An off-line protocol such as NAL is not suitable to prevent large systematic positioning errors for this group (Fig. 2). Although with an extended NAL protocol systematic errors can largely be reduced by correction for gradual time trends during the entire treatment, the daily variation in DIBH and positioning cannot be adequately addressed. This was also confirmed in other studies [8], [10], [16]. The observed intra- and interfraction variability for DIBH treatments might be associated with variations in the respiration cycle involving the composed movements of the abdomen and thorax. As shown in the supplementary data, after patient setup with SGRT in free-breathing, DIBH set-up errors of 15 mm or more may still be observed in CBCT matches.

The addition of SGRT for initial patient positioning did not reduce the observed set-up errors in CBCT matches in our study, nevertheless, an overall more accurate CBCT registration was obtained with SGRT, because the patient’s posture and arm and chin position could already be adjusted a priori based on surface guidance. This contributed to a faster consensus between the technicians during CBCT registration. In addition, SGRT may enable a more patient-friendly workflow in which permanent tattoos can be omitted [23].

SGRT has the advantage that it provides real-time information on the patient’s positioning without the use of ionizing radiation. A reduction in imaging dose can be achieved when applying SGRT for setup in free-breathing as well as in DIBH. Laaksoma et al [16] showed that such a workflow is feasible, although at a cost of a small increase in residual errors. The accuracy of SGRT then still should be verified on a patient-to-patient basis during the first few fractions of the treatment, and routine imaging (e.g., weekly) should be performed to verify consistency of the internal anatomy throughout the treatment course. Therefore, the combination of surface imaging and online imaging remains necessary to ensure accurate dose delivery of DIBH treatments with tight target margins.

Reproducibility, determined from the maximum difference between mean DIBH levels per beam within one treatment fraction, measured on average 1.69 mm (SGRT) and 2.10 mm (OTM). This is consistent with previous studies that reported values between 0.5 mm and 3.4 mm [8], [20], [21], [22], [26], [28]. Visual feedback on the breathing motion, including tolerance levels, further improved the reproducibility to 1.30 mm, like also shown in other studies [22], [29], [30]. In addition, most of the patients appreciated the feedback.

## Conclusion

For accurate radiotherapy breast treatments using a DIBH technique, a daily imaging protocol is required. In combination with online setup correction, non-invasive optical surface guidance systems can provide accurate information on patient positioning prior and during the treatment. Visual feedback to the patient improves patient compliance to DIBH and will facilitate the implementation of gating for this patient group in the near future.

## Declaration of Competing Interest

The authors declare the following financial interests/personal relationships which may be considered as potential competing interests: The department has research collaborations with Elekta AB, Accuray Inc, VisionRT, and Varian Medical Systems.
