# Corrigendum to “Evaluation of image-guided and surface-guided radiotherapy for breast cancer patients treated in deep inspiration breath-hold: A single institution experience” [Tech. Innov. Patient Support Radiat. Oncol. 21 (2022) 51–57]

**DOI:** 10.1016/j.tipsro.2022.12.005

**Published:** 2023-01-07

**Authors:** Joan Penninkhof, Kimm Fremeijer, Kirsten Offereins-van Harten, Cynthia van Wanrooij, Sandra Quint, Britt Kunnen, Nienke Hoffmans-Holtzer, Annemarie Swaak, Margreet Baaijens, Maarten Dirkx

**Affiliations:** Department of Radiation Oncology, Erasmus MC Cancer Institute, Rotterdam, the Netherlands

The authors regret that the following Patient Consent Statement was missing from the above article:

**Patient Consent**

All patients were informed that data can be used for research and QA purposes, and possibly used in scientific publications (anonymized).

  The authors would like to apologise for any inconvenience caused.

