# Supplementary material for: Evaluation of image-guided and surface-guided radiotherapy for breast cancer patients treated in deep inspiration breath-hold: A single institution experience
Source: Tech Innov Patient Support Radiat Oncol. 2022 Feb 17;21:51–7. doi: 10.1016/j.tipsro.2022.02.001 (PMC8861395; doi:10.1016/j.tipsro.2022.02.001)

Supplementary data

SGRT-based positioning of DIBH patients

Figure S1: Boxplot for the observed translations in the three orthogonal directions when positioning based on tattoos and SGRT (blue, 47 patients), or on tattoos only (red, 25 patients). Boxes display the 25%-75% intervals, whiskers are at 5% and 95%, outliers are depicted as crosses.. * denotes a significant difference based on a two-sample t-test (p-value < 0.05).

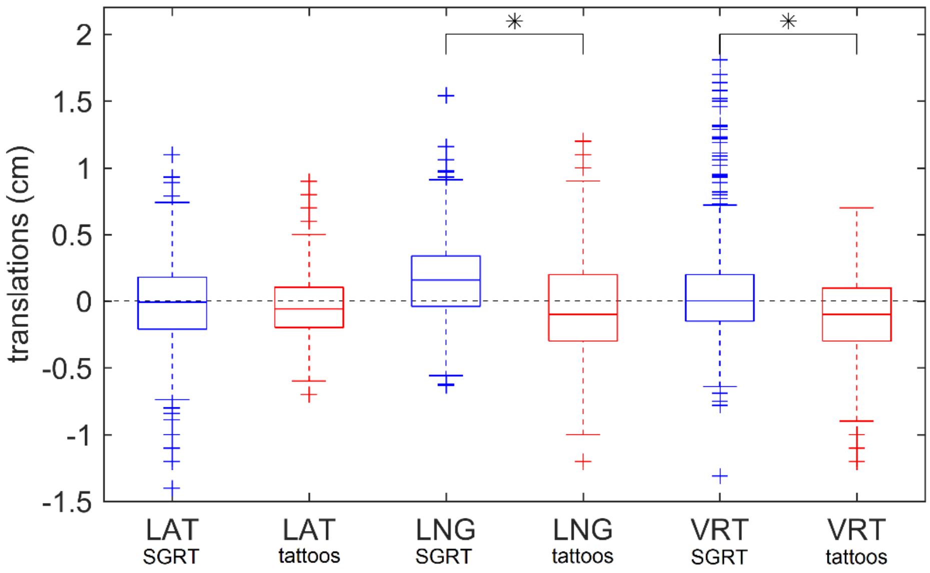

Figure S2: Boxplot for the observed rotations when positioning based on tattoos and SGRT (blue, 47 patients) or on tattoos only (red, 25 patients) Boxes display the 25%-75% intervals, whiskers are at 5% and 95%, outliers are depicted as crosses. * denotes a significant difference based on a two-sample t-test (p-value < 0.05).

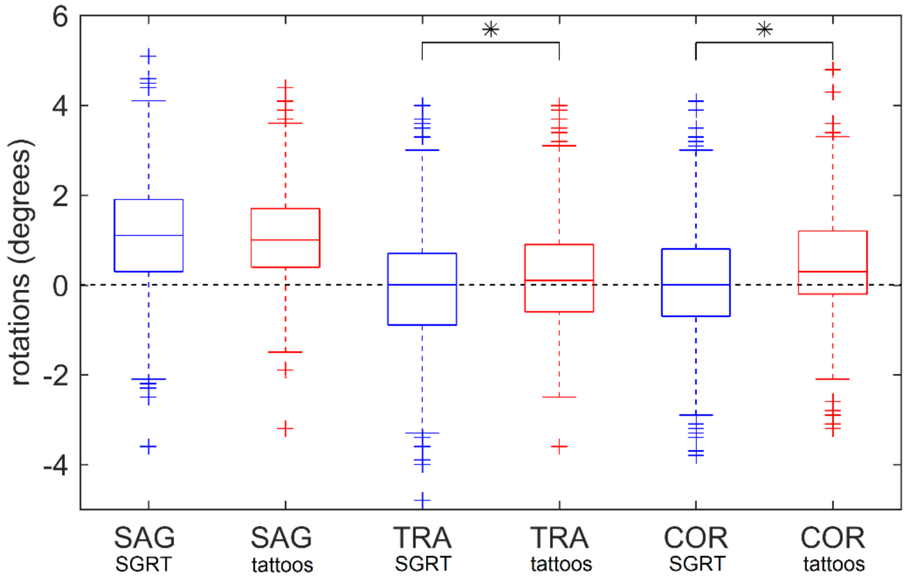

Supplement: Supplementary Data 1 [file mmc1.pdf]
